# The relationship between interoceptive emotional awareness, neuroticism, and depression, anxiety, and stress

**DOI:** 10.1371/journal.pone.0299835

**Published:** 2024-04-16

**Authors:** Natasha Grimble, Jessica Scarfo, Jessica Katherveloo, Michael Ganci, Michelle Ball, Emra Suleyman

**Affiliations:** 1 Institute for Health and Sport (IHES), Victoria University, Footscray, Victoria, Australia; 2 Turner Institute for Brain and Mental Health, Monash University, Clayton, Victoria, Australia; La Sapienza University of Rome, ITALY

## Abstract

COVID-19 has caused challenges to daily living globally, with profound implications for negative mood. A variety of state and trait-based factors can influence how a person may respond and adapt to challenges such as a global pandemic. Personality is an area impacting how a person responds to both internal and external situations (trait) and Emotional Awareness (EA) is a facet of interoception (an awareness of the mind-body connection) that determines the way an individual interprets their physiological state of the body, and the associated emotions (state-trait). Both areas have been well researched in isolation, however the body of literature exploring the relationships between both is much smaller. It would therefore be beneficial to explore the interrelationships of both state and trait factors on wellbeing to enable a more comprehensive understanding. It was hypothesised that EA would moderate the relationship between Neuroticism and Depression, Anxiety, and Stress. Participants residing in Australia during periods of imposed lockdown were included within the study (n = 838; Ages = 18–60 years) and completed an online questionnaire battery including a variety of state and trait questionnaires. A moderation analysis was conducted to explore whether Emotional Awareness changed the relationship between neuroticism and depression, anxiety, and stress utilising an alpha of < .05. EA moderated the relationship between Neuroticism and Anxiety (*p* = .001, 95% CI .03-.17)), and Stress (*p* = .02 95% CI.01-.13), but not Depression (*p* = .23, 95% CI .03-.13). As Neuroticism increased, negative mood increased for all levels of EA, however those high in Neuroticism and EA displayed the highest Anxiety and Stress. Interventions to increase EA, such as mindfulness, may have adverse effects for individuals high in Neuroticism, emphasising the importance of tailored interventions and supporting the assumption that high levels of Neuroticism represent increased vulnerability during a pandemic.

## Introduction

The current study was undertaken in the context of the COVID-19 global pandemic. This pandemic has constituted a social and economic crisis, and created a mental health burden that has been unprecedented in modern times [1,2] A variety of state and trait-based factors can influence how a person may respond and adapt to such challenges. Therefore, the impact of a potentially powerful stressor such as a global pandemic could be moderated through different psychological processes, such as how the threat of illness is reported, and therefore responded to [3]. Essentially, this suggests the effects of COVID-19 on health outcomes may be different for each person according to their psychological predispositions, especially for those at greater risk of experiencing negative mood [4] during the large-scale spread of disease that occurs during a pandemic.

Trait theories describe distinguishing characteristics between people of different personality styles as an explanation for individual differences [5]. These theories focus on the stability and consistency of personality over time, contending that patterns of emotional and behavioural responses remain similar, regardless of environmental stimuli [6]. Individuals can display characteristics of more than one personality type, but generally display an overarching, dominant trait [7]. The most widely used and investigated trait theory is termed the ‘Big-5’ [8]. Within this theory, personality is categorised into five traits including conscientiousness, openness, agreeableness, extraversion, and Neuroticism. These traits are conceptualised to fall on a continuum, with each individual endorsing varying levels of each trait [7]. Of these traits, individuals high in Neuroticism are at most risk of experiencing negative mood relative to the other Big-5 traits [9]. Trait Neuroticism refers to individuals who present as anxious or irritable, and therefore show increased activation in the neural structures associated with sensitivity to threat and punishment [8].

The relationship between personality and mood has been widely explored. It has been suggested that the impact of personality on mood is dependent upon the cognitive demands created by different situations [10]. Highly neurotic individuals tend to react to emotionally arousing stimuli more intensely, and take longer to return to their pre-arousal state [11]. Individuals high in Neuroticism display repeated patterns of negative emotions, rumination, and are more susceptible to chronic stress or other homeostatic imbalances [12]. Strong positive correlations have also been identified between Neuroticism and mood disorders including, Anxiety and Depression [13].

In the real-world, Neuroticism lends a person towards becoming stressed or distressed easily, worrying excessively, and often reacting in a negative way that exceeds the severity of a stimulus [14]. Due to the propensity to react in a negative way, neurotic individuals can face extreme difficulties in reaction to stressful life events or triggers [15] and in ways which may exceed what would be considered a ‘normal’ or typical stress response [16] relative to the other Big-5 traits [17] While Neuroticism appears to have many negative connotations attached, an alternative perspective postulates that it can assist in overcoming barriers. Individuals with high endorsement of this personality trait have been shown to learn better coping mechanisms and strategies [18], possibly due to familiarity with negative emotions. It is proposed that this causes neurotic individuals to respond in a more adaptive way, should they use the neurotic tendencies to motivate and enhance their coping abilities [19]. However, the type of situation, pleasant or unpleasant, can have a significant impact on how a neurotic individual will react [20]. In unpleasant situations there is a much higher likelihood for neurotic individuals to adopt negative response patterns, thereby hindering their ability to respond adaptively to overcome associated barriers. Despite some studies reporting positive effects, it may therefore be concluded that neurotic individuals are at an increased risk of mood disturbance relative to the other Big-5 traits in the context of a global pandemic.

An often-neglected angle of the state-trait perspective of vulnerability to experiencing Depression, Anxiety or Stress is the role of the body, or awareness of the mind-body connection. The body’s physiological systems are constantly fluctuating in the attempt to maintain homeostasis [20]. The ability to perceive one’s own physiological state including muscular and visceral sensations, and emotional and cognitive experiences is known as interoception [21–23]. Interoception is an umbrella term with a focus on recognition of the internal bodily state, however it has emerged as a multi-faceted concept. For example, Emotional Awareness (EA) is a construct that represents the emotions attributed to a person’s physiological state [24] that can be differentiated from experiences of fear and worry due to unfamiliarity or irritation related to bodily sensations. Although the EA concept is similar to general emotions, there is a greater emphasis on unconscious emotional attribution to physiological state, rather than internal (thoughts) or external triggers [25]. Unsurprisingly, in literature there is a positive relationship between mood (a general ‘feeling’ without an explicit cause) and emotion (short lived expressions with a known cause [26,27]. This extends to EA which has been found to influence mood and to predict psychopathology [28].

A technique often prescribed to correct difficulties with emotional awareness is Mindfulness. Mindfulness refers to an awareness and acceptance toward one’s present state encompassing feelings, thoughts, and bodily sensations [29]. Importantly, Interoceptive awareness (IA) is often used synonymously with mindfulness and has been shown to have links to many positive health outcomes, and to assist in overcoming daily issues [30]. Due to this, individuals may be encouraged to employ mindfulness techniques in an attempt to increase IA. However, research has also suggested that being highly aware of physiological states can indicate dysfunction. This may be because mood disorders and states such as Depression and Anxiety can be associated with negative self-referential processing [31]. Therefore, individuals who regularly reflect on their physiological state may engage in self-evaluation processes which have the ability to trigger maladaptive cognitive reactivity. For individuals who have a mood that is consistently negative there may also be an inability to disengage, which in turn can perpetuate or increase their dysfunction. Conversely, research has also indicated that high IA can cause downregulation of affect related arousal. This implies that high IA assists in regulating emotions in response to negative stimuli [32]. Therefore, IA may increase Depression, Anxiety or Stress for some individuals, but decrease it for others, thereby highlighting the importance of individual differences.

The implications of COVID-19 have been extremely challenging due to the unprecedented social and economic changes, and the huge burden on mental and physical health it has wrought [33]. The concept of health anxiety refers to feeling concerned about becoming unwell or contracting a severe illness, while being objectively healthy [34]. In some circumstances this fear is severe and results in hypochondriasis [35]. For individuals who already experience feelings of distress surrounding their health, as is likely to be seen in neurotic individuals, a global pandemic is likely to exacerbate these worries and increase experiences of negative mood. It is also important to note that health anxiety can be the result of high IA [36] as the result of stronger internal bodily signals or higher allocation of attentional resources to internal sensations [36]. In other words, higher IA may lead to increased hypervigilance to bodily sensations that may be experienced [34]. Therefore, the relationship between Neuroticism and mood in the context of a global pandemic, could be cause for concern, particularly for people high in EA.

This study aimed to explore the relationship of Neuroticism on Depression, Anxiety and Stress given levels of EA to address the gap in knowledge surrounding the influence of the mind-body connection. Specifically, as there is a known relationship between Neuroticism and negative mood outcomes, it is important to explore the association of the awareness of physiological state (EA) on this relationship, as mindfulness techniques continue to gain popularity in society, and are easily accessible in any context, including that of a global pandemic. It was hypothesised that EA would moderate the relationship between Neuroticism, Depression, Anxiety and Stress.

## Materials and methods

### Participants

A total of 838 participants (451 females, 385 males and 1 transgender; Mean age = 29.54 years, SD = 9.88) who resided Australia were recruited by use of convenience and snowball sampling techniques between the 29^th^ of April 2020 to the 29^th^ of April 2022, during various stages of COVID-19 lockdowns. The completion rate of the questionnaires in the present sample was 93.5% Inclusion criteria for this study stipulated participants were aged between 18–60 (inclusive), spoke fluent English, and were not currently taking medication for mental health conditions. Anyone who did not meet these criteria’s were excluded.

### Measures

#### The mini IPIP scale of personality

The Mini IPIP Scale of Personality (IPIP; [37]) is a 20-item scale that measures the Big-5 Personality Traits. The scale uses a 4-point Likert scale ranging from 1 (strongly agree) to 4 (strongly disagree), with a score range of one to 16. The IPIP has displayed psychometric properties that are comparable to other personality assessments of the Big-5, despite being much shorter than traditional scales. Specifically, reliability coefficients of each of the five traits include, Extraversion (α = .77), Agreeableness (α = .70), Conscientiousness (α = .69), Neuroticism (α = .68) and Intellect/Imagination (Openness; α = .65; [38]). The IPIP has been widely used in research with high content, convergent and criterion validity [39].

#### The multidimensional assessment of interoceptive awareness -version 2

The Multidimensional Assessment of Interoceptive Awareness (MAIA-2; [25]) is a self-report questionnaire using a Likert-scale pertaining to how often each of 37 statements applies to a participant ranging from 0 (never) to 5 (always). The MAIA-2 has been reported to demonstrate strong content, convergent and criterion validity [40]. The questionnaire yields 8 state-trait based subscales, with high reliability scores, namely Noticing (α = .69), Not-distracting (α = .66), Not-worrying (α = .67), Attention Regulation (α = .87), Emotional Awareness (α = .82), Self-Regulation (α = .83), Body Listening (α = .82), and Trusting (α = .79).

#### Depression, anxiety and stress scale– 21

The DASS-21 [41] is a 21 item, self-report questionnaire measured on a four-point Likert scale ranging from 0 (did not apply to me at all) to 3 (applied to me very much, or most of the time), with a score range of 0–21. Seven of the 21 items in this scale apply to each domain namely Depression, Anxiety or Stress. This questionnaire is frequently used in research, displaying excellent psychometric properties, with reliability scores ranging from α = .87 to α = .94 and; strong content, convergent and criterion validity [42].

### Procedure

Ethical approval was sought and granted by the Victoria University Human Research Ethics Committee–application ID HRE21-158. The current study was part of a larger project investigating the psychological factors that influence perceived vulnerability to infectious disease during the COVID-19 pandemic. Data was collected using the Inquisit software program (Version 6) created by Millisecond®. Prior to participants commencing the study they provided informed, written consent by ticking a check box noting ‘I consent’. They were then directed to the test battery comprising of 18 tests assessing psychological, physiological, and cognitive domains, requiring up to 75 minutes for completion. Counterbalancing was used by providing three alternative orders of the battery to minimise the impact of dropout part-way through. Data was downloaded and analysed using the Statistical Package for the Social Sciences (SPSS) version 26.

### Statistical design

A moderation analysis was selected for the present study to explore whether EA altered the relationship between Neuroticism and wellbeing outcomes. To ascertain sample size a power samples test was conducted and revealed for *p =* .*05*, effect size *f* ^2^ = .15 the required sample size is 107. Therefore, the sample size in the present study is adequately powered (n = 838). All measures used in the study, namely Emotional Awareness (α = 0.86), Neuroticism (α = 0.71), Depression (α = .93), Anxiety (α = .83) and Stress (α = .86) displayed high reliability scores in this sample. The sample was not screened for those individuals with high Neuroticism only, as each of the Big-5 personality traits are not binary or categorical (present or absent) in an individual, but rather endorsed to varying levels reflecting a continuum of high or low. The relationship between personality and mood has been widely explored, however the relationship of the physiological state of the body, and level of awareness of associated emotions has not been explored. For this reason, EA was chosen as the moderator in the present study. It is also important to note that the chosen analysis was a moderation opposed to other highly powered statistical measures such as Structural Equation Modelling as the outcome variables (Depression, Anxiety and Stress) are distinct, individual constructs [41]. Moreover, the present study was not trying to identify latent constructs and their relationships to one another as each scale used is pre-existing and retains strong reliability and validity in past research and the current sample, therefore the analysis chosen was the best fit [43]. During the data screening and cleaning process data was screened for missing values. Any indicators with missing values were excluded from analysis. Outliers were also screened and excluded from analysis. All assumptions were then checked (see ‘assumptions’ below).

#### Assumptions

All assumptions pertinent to moderation analysis were checked and met for each individual analysis. The data for all analysis satisfied the assumption of normality by means of skewness and kurtosis, as values fell within acceptable ranges of +3 to -3 [44]. Linearity was assessed by a correlation matrix which displayed significant correlations between the predictor and dependent variables. The Durbin-Watson statistic fell within the acceptable ranges of 1–3 therefore satisfying the assumption of independence of errors. Tolerance was above 0.1 and VIF was below 10 therefore nil issues with multicollinearity were detected. With the critical χ2 set at 18.48 no multivariate outliers were detected as the maximum Mahalanobis distance did not exceed the critical value.

## Results

Table 1 displays the descriptive statistics for the variables Depression, Anxiety Stress, EA, and Neuroticism.

**Table 1:** Means and standard deviations for key variables.

	*n*	*M*	*SD*
Depression	838	7.06	5.44
Anxiety	838	5.30	4.29
Stress	838	7.79	4.44
Emotional Awareness	838	3.36	1.02
Neuroticism	838	11.78	3.22

### Analysis one—depression

Moderation analysis using Haye’s PROCESS indicated a good model fit to the data, with Neuroticism and EA together accounting for 33% of the variance in Depression, *F*(3,834) = 138.54, *p* < .001. Coefficients for the regression model can be seen in Table 2.

**Table 2:** Coefficients for the regression model with neuroticism and emotional awareness predicting depression.

Model	Coeff	SE	*t*	*p*	LICI	ULCI
Neuroticism	.98	.05	20.33	.001	.88	1.07
EA	-.37	.15	-2.43	.02	-.67	-.07
Neuroticism x EA	.05	.04	1.19	.23	.03	.13

**Fig 1:**
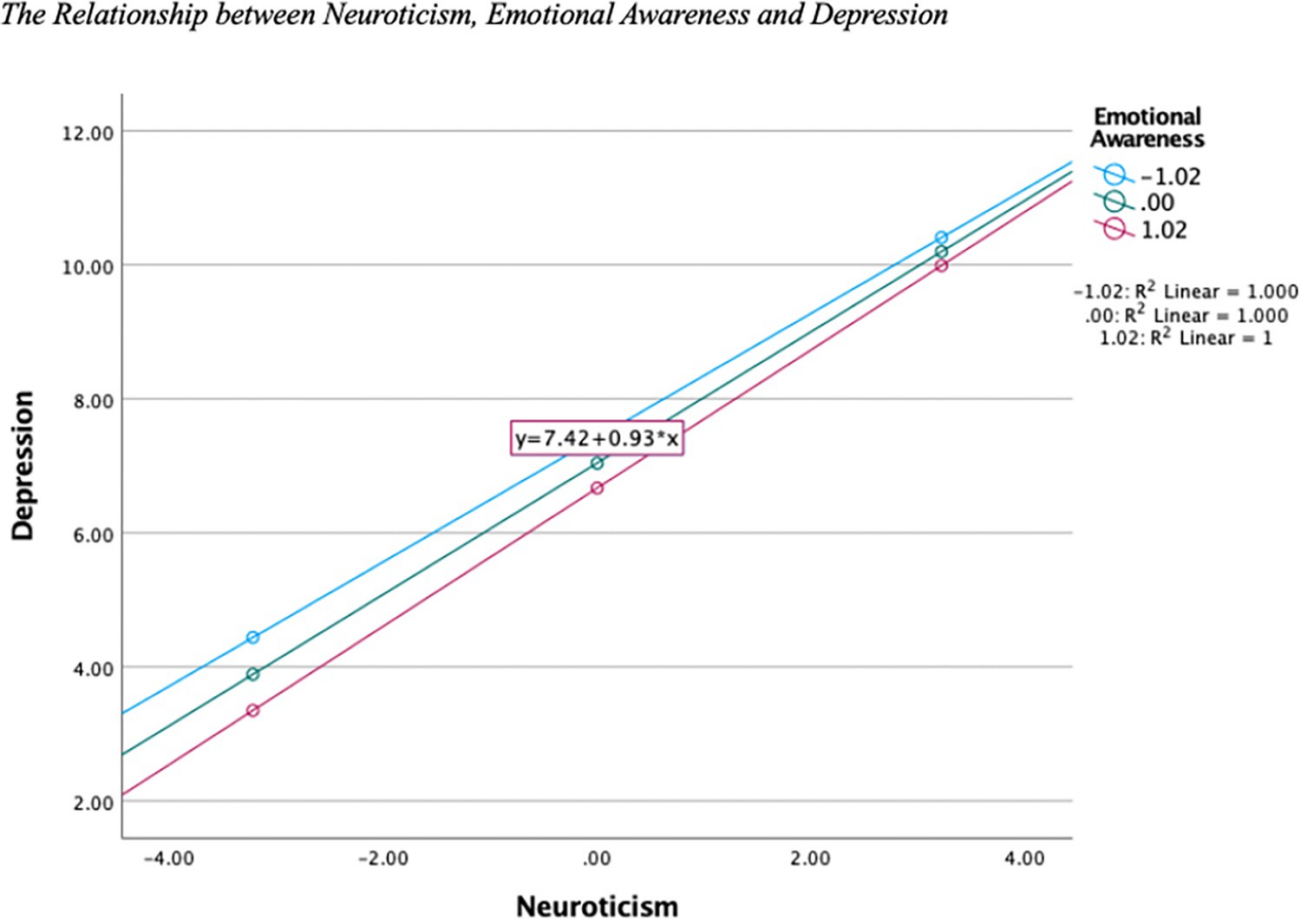
The moderating effect of emotional awareness on the relationship between neuroticism and depression.

Neuroticism was a positive predictor, and EA a negative predictor of Depression. Fig 1 displays that as neuroticism levels increased, so did experiences of depressed mood for all levels of EA. No interaction effect was identified.

### Analysis two—anxiety

Moderation analysis using Haye’s PROCESS indicated a good model fit to the data, with Neuroticism and EA together accounting for 29% of the variance in Anxiety, *F*(3,834) = 111.35, *p* < .001. Coefficients for the regression model can be seen in Table 3.

**Table 3:** Coefficients for the regression model with neuroticism and emotional awareness predicting anxiety.

Model	Coeff	SE	*t*	*p*	LICI	ULCI
Neuroticism	0.64	.04	16.22	.001	.56	.71
EA	0.74	0.12	5.97	.001	.50	.98
Neuroticism x EA	0.10	.03	2.83	.001	.03	.17

**Fig 2:**
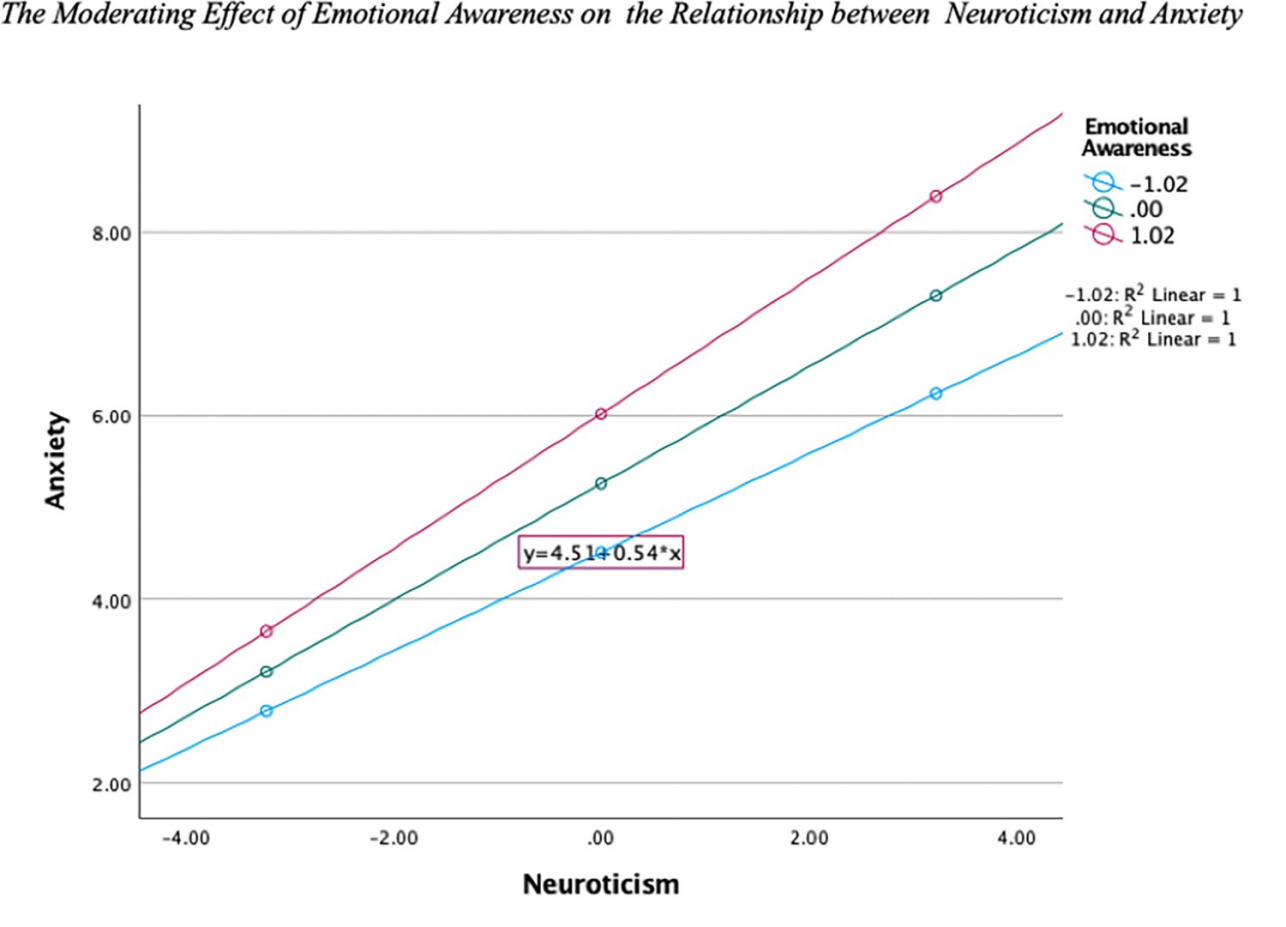
The moderating effect of emotional awareness on the relationship between neuroticism and anxiety.

Both Neuroticism and EA were positive predictors of Anxiety. There was also a significant interaction of Neuroticism and EA on Anxiety, indicating a moderation effect. Fig 2 shows that as levels of Neuroticism increased, so did the experience of Anxiety for all levels of EA, with the highest level of Anxiety seen in individuals with high levels of Neuroticism and EA.

### Analysis three—stress

Moderation analysis using Haye’s PROCESS indicated a good model fit to the data, with Neuroticism and EA together accounting for 46% of the variance in Stress, *F*(3,834) = 235.61, *p* < .001. Coefficients for the regression model can be seen in Table 4 below.

**Table 4:** Coefficients for the regression model with neuroticism and emotional awareness predicting stress.

Model	Coeff	SE	*t*	*p*	LICI	ULCI
Neuroticism	.90	.04	68.16	.001	.83	.97
EA	.48	.11	4.28	.001	.26	.70
Neuroticism x EA	.07	.03	2.33	.02	.01	.13

*Note*: Outcome variable = Stress.

**Fig 3:**
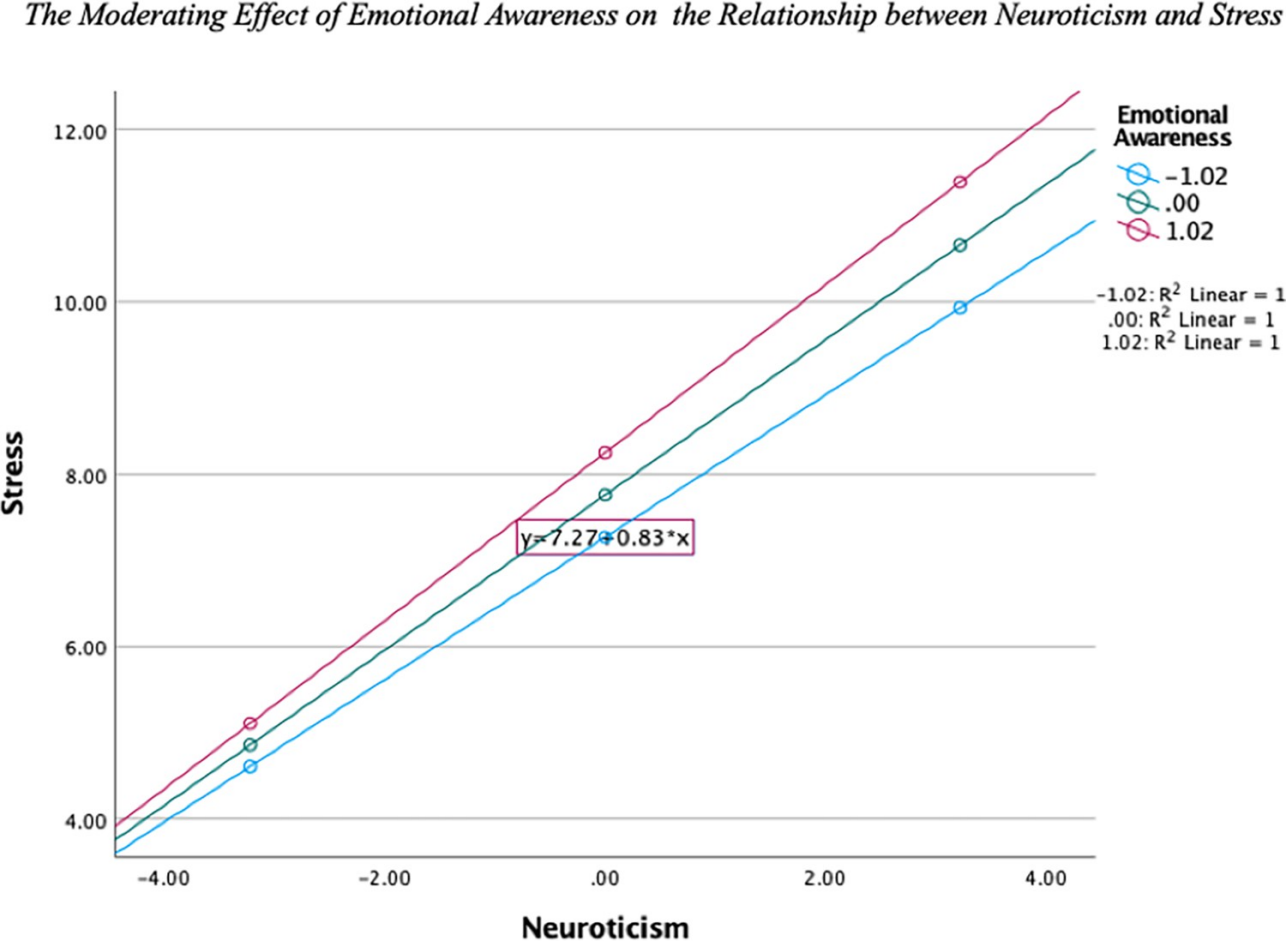
The moderating effect of emotional awareness on the relationship between neuroticism and stress.

Both Neuroticism and EA were positive predictors of Stress. The association between Neuroticism and Stress was moderated by EA as indicated by the significant interaction. Fig 3 shows that for all levels of EA, Stress increased as levels of Neuroticism increased, with the highest levels of Stress seen in individuals with high levels of both Neuroticism and EA. This relationship can be seen in Fig 3.

## Discussion

The present study aimed to investigate the relationship between Neuroticism and Depression, Stress, and Anxiety given levels of EA. The hypotheses that that there would be a moderating effect of EA on the relationship between Neuroticism and Anxiety (analysis two) and Stress (analysis three) were supported, and the hypothesis that that there would be a moderating effect of EA on the relationship between Neuroticism and Depression was not supported.

The moderation analysis found that individuals with high levels of EA and high Neuroticism scores displayed the highest Anxiety and Stress scores. There was no interaction effect of Neuroticism and EA on Depression, however high levels of Neuroticism indicated higher Depression. In the context of a global pandemic, individuals are likely to feel anxious or stressed, for a variety of reasons [45]. With the potential chance of contracting COVID-19, it is possible that individuals were more likely to attribute feelings of worry, uncertainty or apprehension to their physiological state, in addition to social restrictions, the saturated media coverage of the pandemic changed behaviours including panic buying, arguably enhancing fear responses and concern for contracting the illness. It is therefore unsurprising that as levels of Neuroticism increased, so did experiences of Depression, Stress and Anxiety. However, the interaction effect between EA and Neuroticism is interesting, as research often indicates that IA is a positive tool for emotional regulation and overcoming life barriers. While this finding is true for individuals who have low levels of Neuroticism relative to the other Big-5 traits, it is untrue for those at the higher end of the Neuroticism spectrum, as demonstrated by current findings. Those with both high EA and Neuroticism demonstrated higher Anxiety and Stress and is consistent with literature indicating that that being highly aware of physiological state can cause intense feelings of Anxiety, Stress and overall negative mood [15], but contradicts research suggesting that high EA is a positive tool to reduce low mood.

Stressors will always be present in daily life at differing levels for all individuals, and the incidence of a global pandemic inevitably adds an additional layer of Stress and Anxiety in the general population [46]. When considering the relationship between EA and Neuroticism without the addition of COVID-19, research suggests that high levels of EA can either be positive or negative. As the current study supports both directions, the level of Neuroticism becomes extremely important as it will determine the implications of varying levels of EA. Due to both the behavioural elements and cortical activation seen in the limbic system in neurotic individuals, the finding that the relationship between Neuroticism and mood changes as a function of EA has interesting implications for psychological research and practice. Research predominantly suggests that an individual’s underlying personality trait should determine their emotional response patterns, and thus mood reactivity. However, the present study indicates that mood is determined by the interaction of state (EA) and trait factors, as opposed to just an individual’s trait-based response patterns. This pattern was shown to apply to Stress and Anxiety levels, but not for Depression. It is possible that this is related to the context of the global COVID-19 pandemic, where levels of Anxiety and Stress related to health and changed life circumstance were likely to be particularly relevant. Most importantly, this interaction highlights that alternative approaches to treatment should be taken for individuals based on their trait-based Neuroticism levels.

A noteworthy finding on the present study was the finding of a positive relationship between EA and anxiety and stress, while the inverse was found for depression. This finding is consistent with studies who have also identified that as levels of depression increase, levels of EA decrease [47] However, contrasts studies suggesting both anxiety and depression maintain the same direction [48]. The difference in direction may be explained as while each of these three negative emotional states share similar underlying mechanisms and activation of the hypothalamic-pituitary-adrenal (HPA) axis, the way in which they are regulated varies [49]. Anxiety and stress entail increases in various physiological bodily responses whereas depression shows a decrease. This is also consistent with the presentation and symptomology associated with each emotional state. In anxiety individuals experience elevation in many forms including heart rate, worry or nervousness, similarly stress can cause an elevated state of mental worry [50]. While depression shows many similarities, it can also case a reduction in energy. In the context of a global pandemic, it is likely that having a high level of awareness toward the emotional attribution toward the physiological state of the body increases stress and anxiety (positive relationship) due to health anxiety or fear of contracting an illness. Moreover, as these are feelings of elevation, being more aware of emotions and their associations to the physiological state of the body may contribute to increased feelings of worry. Conversely, as depression surrounds feelings of low mood and a reduction in energy, being aware of the body (and its emotions) may enable the regulation of low affect, but not to reduce the elevation associated with stress and anxiety. While this may be a potential explanation to these findings, they contradict studies that have identified interoceptive function to be impaired in depression [51]. However, it is important to note that interoception is a multifaceted construct, and further exploration toward the individual facets and their relationship with wellbeing outcomes may be required to understand if they operate in different ways, which may also explain this finding as studies often do not explore the MAIA facets in isolation [23,47,48]. In a similar vein, a strength of the present study is that depression, anxiety, and stress were explored as individual facets. As each mood disposition is distinctly different it is important that they be explored in isolation for a more comprehensive understanding.

The results of the current study imply that interventions should be tailored to the specific emotional state an individual is facing, as the relationship between Neuroticism, Stress and Anxiety differs to Depression. This finding has particular implications for mindfulness-based interventions which focus on increasing the awareness of the physiological state, as it suggests that being mindful may not always be a positive tool for mood maintenance. In general, mindfulness techniques have been shown to increase IA [52] and allow individuals to disengage with maladaptive behaviour and emotional response patterns. In some instances, mindfulness can also assist in reducing rumination towards negative stimuli [53]. Furthermore, research has found that mindfulness can also reduce the impact of neurotic thought processes [54]. The findings of the present study suggest that for individuals low in Neuroticism, mindfulness practises may be beneficial for improving and maintaining low experiences of Anxiety and Stress through the enhancement of EA. However, for individuals higher in Neuroticism, the present study suggests that focusing on becoming more in tune with one’s emotional attribution to their physiological state may not have the same protective relationship over negative mood outcomes of Stress and Anxiety, and may in fact have the reverse effect.

Mindfulness techniques have received large amounts of support in both the lay and scientific communities as an easy to administer intervention that promotes improvement of self-awareness and overall wellbeing, however current practice has not considered if changes need to be made due to the COVID-19 crisis. It seems that the current climate of generalised Anxiety instilled due to the pandemic may change the association between EA and mood such that fears and anxieties may become exacerbated if there is a fixation on changes that may indicate physiological imbalances [6], at least for individuals high in Neuroticism. For these individuals the experience of change to their physiological state may exaggerate concern connected to fears of contracting COVID-19. Furthermore, if an individual is highly aware of their physiological state, they may be constantly drawn to noticing these changes since homoeostasis is a continual process [55], thus producing the threat of higher experiences of Anxiety or Stress.

The maintenance of mental health has become more recognised in Western societies as a crucial factor for maintaining overall health and wellbeing. Due to progressions in technology and large influence of social media, individuals often seek advice and trends on how to maintain their mental wellbeing from these types of sources [56]. A large body of research has demonstrated the positive impacts of regular physical activity, maintaining a healthy diet and adequate rest for the maintenance of mood [57] and has suggested mindfulness-based interventions as extremely useful towards this end [58]. The present study has supported the notion that mindfulness can be a positive tool for improving or maintaining mood, however it has also contributed to knowledge by suggesting that mindfulness is not a one-size fits all technique that is helpful in all circumstances.

### Limitations and future directions

Future research may wish to explore a sample with clinical populations to gain a better understanding of the applicability of findings. The present study did not extend to examine the remaining four personality types. Some authors have suggested that Neuroticism, extraversion, and conscientiousness may moderate depressive symptomology [53]. Mechanisms of this action are as yet unknown, however results indicate that a combination of low extraversion, low conscientiousness and high Neuroticism have a link to depressive symptomology, as well as other forms of psychopathology in both healthy and clinical populations. It would, however, be beneficial for the interaction between personality traits and EA to be explored further to create a framework for tailoring interventions for Anxiety and Stress based on personality profiles, including after the impact of COVID-19 has passed. Likewise, future research could also explore the relationship between personality and Depression, Anxiety and Stress in relation to other dimensions of IA. Future research may also wish to utilise methods other than cross-sectional to explore causation and mitigate the inherent bias found in self-report questionnaires. Finally, recruitment via the online recruitment service–Prolific captures participants with access to the internet with the incentive of monetary compensation for participation. This demographic may therefore not fully encapsulate the diversity of attributes and perspectives found in the wider population, impacting the representativeness of the current findings. It is suggested that future research may wish to seek participants from various settings including those with a range of experience with mindfulness-based practice to verify if the patterns observed in the present study persist. This will enable a more nuanced understanding of the relationship between personality, EA and wellbeing.

## Conclusion

The interaction between Neuroticism and EA on Anxiety and Stress, suggests that increasing IA of emotions attached to a person’s physiological state is a useful tool for individuals who endorse lower levels of Neuroticism, but the opposite is true for highly neurotic individuals, at least in the context of a global pandemic. The results of the study demonstrate that interventions, such as mindfulness, that are designed to increase a person’s emotional awareness may actually increase distress in people who are higher on the Neuroticism spectrum. It is therefore, important to develop targeted interventions to assist in maintaining and improving mental and physical wellbeing. Therefore, it is suggested that clinicians recommending mindfulness, should first assess levels of neuroticism, and where relevant aim to reduce a person’s level of neurotic responses *before* introducing mindfulness as a technique to support a healthy mood. Overall, the study indicates that to improve health and wellbeing during a global pandemic, interventions may need to be tailored for some individuals, particularly those who endorse high levels of Neuroticism.

## Supporting information

S1 Table Socio-demographic information. (DOCX)
