# Supplementary material for: The relationship between interoceptive emotional awareness, neuroticism, and depression, anxiety, and stress
Source: PLoS One. 2024 Apr 16;19(4):e0299835. doi: 10.1371/journal.pone.0299835 (PMC11020380; doi:10.1371/journal.pone.0299835)
Supplement: S1 Table — (DOCX) [file pone.0299835.s001.docx]

**S1 Table: Sociodemographic Information**

| Variable | | |
| --- | --- | --- |
|  | N | % |
| Sex |  |  |
| Male | 414 | 46.2 |
| Female | 481 | 53.6 |
| Transgender | 1 | .1 |
| Non-binary | 1 | .1 |
| State of Residency |  |  |
| Victoria | 347 | 50.2 |
| New South Wales | 165 | 23.9 |
| Australian Capital Territory | 14 | 2.0 |
| Queensland | 81 | 11.47 |
| South Australia | 37 | 5.4 |
| Western Australia | 34 | 4.9 |
| Tasmania | 13 | 1.9 |
| Covid Diagnosis |  |  |
| Yes | 2 | .2 |
| No | 892 | 99.8 |
| Family Covid Diagnosis |  |  |
| Yes | 34 | 3.8 |
| No | 860 | 96.2 |
| Level of Education |  |  |
| Year 10 or below | 10 | 1.4 |
| Year 12 | 165 | 23.4 |
| TAFE Vocational Training | 57 | 8.1 |
| Graduate Diploma | 21 | 3.0 |
| Graduate Certificate | 43 | 6.1 |
| Bachelor’s degree | 224 | 31.8 |
| Honours Degree | 48 | 6.8 |
| Master’s Degree | 106 | 15.1 |
| PhD or Doctorate | 30 | 4.3 |
| Pandemic Job Loss |  |  |
| Yes | 98 | 13.9 |
| No | 497 | 70.6 |
| Not applicable | 109 | 15.5 |
| Professional Field |  |  |
| Manager | 43 | 6.1 |
| Professional | 220 | 31.3 |
| Technician and Trade Worker | 30 | 4.3 |
| Community and Personal Service Worker | 49 | 7.0 |
| Clerical and Administrative Worker | 79 | 11.2 |
| Sales Worker | 65 | 9.2 |
| Machinery Operator and Driver | 4 | .6 |
| Labourer | 23 | 3.3 |
| Unemployed | 191 | 27.1 |
| Sports Engagement |  |  |
| Yes | 632 | 70.7 |
| No | 262 | 29.3 |
